# Bliss in and Out of the Body: The (Extra)Corporeal Space Is Impervious to Social Pleasant Touch

**DOI:** 10.3390/brainsci11020225

**Published:** 2021-02-12

**Authors:** Chiara Spaccasassi, Ivana Frigione, Angelo Maravita

**Affiliations:** 1 Centre for Studies and Research in Cognitive Neuroscience, ‘Alma Mater Studiorum’, Department of Psychology, Cesena Campus, Bologna University, 47521 Cesena, Italy; 2 Department of Psychology, University of Milano-Bicocca, Piazza Ateneo Nuovo 1, 20126 Milano, Italy; i.frigione@campus.unimib.it (I.F.); angelo.maravita@unimib.it (A.M.); 3 Milan Centre for Neurosciences, 20126 Milano, Italy

**Keywords:** affective touch, body ownership, peripersonal space, visuo-tactile, rubber hand

## Abstract

Slow, gentle stimulation of hairy skin is generally accompanied by hedonic sensations. This phenomenon, also known as (positive) affective touch, is likely to be the basis of affiliative interactions with conspecifics by promoting inter-individual bindings. Previous studies on healthy humans have demonstrated that affective touch can remarkably impact behavior. For instance, by administering the Rubber Hand Illusion (RHI) paradigm, the embodiment of a fake hand enhances after a slow, affective touch compared to a fast, neutral touch. However, results coming from this area are not univocal. In addition, there are no clues in the existing literature on the relationship between affective touch and the space around our body. To overcome these lacks, we carried out two separate experiments where participants underwent a RHI paradigm (Experiment 1) and a Visuo-Tactile Interaction task (Experiment 2), designed to tap into body representation and peripersonal space processing, respectively. In both experiments, an affective touch (CT-optimal, 3 cm/s) and neutral touch (CT-suboptimal, 18 cm/s) were delivered by the experimenter on the dorsal side of participants’ hand through a “skin to skin” contact. In Experiment 1, we did not find any modulation of body representation—not at behavioral nor at a physiological level—by affective touch. In Experiment 2, no visuo-tactile spatial modulation emerged depending upon the pleasantness of the touch received. These null findings are interpreted in the light of the current scientific context where the real nature of affective touch is often misguided, and they offer the possibility to pave the way for understanding the real effects of affective touch on body/space representation.

## 1. Introduction

Since Aristotle’s classification within the five main senses, touch has been considered crucial to explore the external world effectively. In our daily life, we often experience the phenomenon to be “touchant-touchè” [1], that is to actively or passively interact with the physical environment, respectively. The former rests on the exteroceptive cutaneous sense of touch which allows to identify and discriminate manipulated objects (e.g., texture, shape). The latter, instead, is intimately linked to the interoceptive sense of touch that is also responsible for the emotional characterization of being touched (e.g., skin to skin social contact) [2]. Despite some of its exteroceptive ingredients, the so-called ‘affective touch’, which is a gentle and subtle skin stroking accompanied by hedonic sensations, largely falls within this latter category [3].

In recent years, an ever-growing mole of studies on affective touch has been conducted. To be affective, the stroking should be delivered in the hairy skin (e.g., the dorsal side of the hand) with a constant low speed [4,5,6]. These peculiarities should trigger the activation of C-tactile afferents (CTs), a system of unmyelinated nerve fibres that seems to be responsible for the pleasure sensation that accompanies this kind of touch [4,7]. Indeed, along with being susceptible to the sensory features (e.g., roughness) of the stroking [8], CTs respond most vigorously when the touch is delivered at intermediate velocity (1–10 cm/s) [4]. Moreover, the stroking delivered at these optimal velocities is also subjectively experienced as the most pleasant [4].

CTs fire optimally at skin temperature, so they are supposed to be tuned to human affective caress [9]. Unsurprisingly, the features characterizing this light touch are akin to those adopted during mother–infant interactions and, more generally, they recall the body contact repertoire usually flaunted when interfacing with loved ones [7]. There is, indeed, a substantial agreement in assuming that CTs’ fibres are tuned to affiliative behaviours with the final aim to promote interpersonal bonds with conspecifics [10]. Notably, the type of stroking and the context where it is delivered are crucial factors in determining the valence of the touch. Indeed, stimulations conveyed by rubber bands [11] could lead to the experience of an unpleasant touch even when the typical parameters for the induction of the affective touch are respected. Despite its possible negative valence, most of the studies on affective touch has been focussed on its positive side and, therefore, only the pleasant touch will be considered from now on. 

The existing literature on healthy adults frequently reports this hedonic side of affective touch both through self-report explicit measures (e.g., questionnaire using Visual Analogue Scale, Likert Scale as response modality) [5,12,13,14,15] and implicit parameters (e.g., skin temperature, electromyographic activity) [12,15,16,17,18,19]. Not surprisingly, a conspicuous mole of evidence from neurophysiology clearly reported a projection of CT-afferents to the emotional system [3,20]. To highlight how this kind of touch is intimately linked to the rewarding-emotional circuitry, a recent neuropsychological study clearly demonstrates that damage to posterior and anterior insula—a critical region for autonomic function [21] and addiction regulation [22]—reduces tactile, contralateral and ipsilateral pleasantness sensitivity, respectively [23].

Right on the heels of this scientific enthusiasm, recently an increasing body of empirical evidence has been collected on the efficacy of this gentle touch in modulating human behavior and cognition. At present, body representation is undoubtedly one of the most prominent neuroscientific and prolific fields of research. In particular, body ownership—the feeling that your body belongs to you [24]—turned out to be malleable to the hedonic touch. Indeed, several studies, demonstrated that a slow touch on hairy skin can enhance body ownership, as highlighted by the more substantial proprioceptive drift [12] and self-report embodiment questionnaire responses [25,26] recorded during the rubber hand illusion paradigm (RHI) [27]. RHI is commonly used to study body ownership, and it consists of stroking with spatial and temporal synchrony the real hidden hand of participants together with an anatomical compatible and visible rubber hand. Along with reporting subjective sensations that the fake hand belongs to them, participants also show a shift in the perceived location of their stimulated hand toward the rubber hand (i.e., proprioceptive drift), generally interpreted as an index of successful illusion (for a different perspective, see [28,29,30]. Curiously, the aforementioned studies about RHI and affective touch, found complementary results: in the first one, the objective measure of embodiment (i.e., proprioceptive drift) but not the subjective one (i.e., questionnaire) was altered by affective touch, while in the second and third study the reverse situation occurred. The scenario becomes even more critical when taking into accounts other experimental paradigms sensitive to body ownership. For instance, de Jong et al. [31] found that, by means of a virtual reality full-body illusion, affective touch was effective to enhance body ownership only in the first half of experiments that they carried out. Still, it did not affect the remaining other half. Another relevant critical point comes from implicit measures used to characterize affective touch. Indeed, heartbeat and electromyographic activity recorded from zygomatic muscle turned out to be significantly affected in some cases [26,32] but not in others [19], by the gentle touch. Again, electrodermal activity is another disputed index, as revealed by contrasting results already present in the literature. In particular, higher skin conductance response (SCR) for neutral rather than affective touch has been reported by [16] and [33], thus suggesting the soothing effect of soft stroking. However, when comparing three different kinds of touch—among which only one was affective—delivered at three different velocities (0.3 cm/s vs. 3 cm/s vs. 30 cm/s), Ref. [34] showed that only the fastest touch was connected to higher SCR than the other two, while no difference was recorded between the slowest and the affective one. To summarize, all of these discrepancies wrap the role of affective touch on body representation in the uncertainty and call for more research in this field.

Furthermore, there is no evidence of whether affective touch could affect the perception of the space immediately surrounding the body, the so-called “Peripersonal Space” (PpS), which seems largely linked to body representation [35]. It is widely known that PpS is exquisitely connected to our body, so much that authors are still wondering whether it makes sense using two labels (i.e., PpS and Body Schema) that likely refer to the same concept [35]. Indeed, we traditionally conceive PpS as the space directly surrounding our body [36,37], which serves as a privileged interface between the body and the external world [36]. In accordance with this point of view, electro-physiological studies on monkey brain [38] and neuroscientific research in humans [39,40] showed that multisensory integration processes are gradually more powerful when they occur closer to the body. In particular, visuo-tactile interactions seem to be particularly sensitive to the spatial distance at which visual stimuli are positioned while the concurrent tactile input is delivered [39,41,42]. Indeed, using a visuo-tactile interaction task (VTI) where looming visual stimuli are presented simultaneously with tactile ones, the above-mentioned studies revealed that response to tactile input is faster when the concurrent visual image is located close to rather than far from the body. This is compatible with the presence in our brain of a fronto-parietal network that seems to be a relevant neural underpinning of PpS representation [36,38]. The PpS system contains bimodal (visuo-tactile) cells whose firing rate becomes even more massive when the looming visual stimulus gets closer to the body [43]. This behavior is explained by their own configuration showing a visual receptive field that matches the location of tactile stimuli on the body surface, remaining anchored to it even when the latter moves [36]. Surprisingly, under specific circumstances, the visual properties of these neurons can be extended to a portion of space beyond that covered by the tactile receptive field [38]. Indeed, these visuo-tactile interactions in space turned out to be sensitive to contingent factors, such as the visual stimulus valence connotation [39,44] as well as anxiety [45]. However, how spatial visuo-tactile interactions could be shaped by the different types of touch (i.e., affective touch) has never been investigated. At the same time, it could be a further relevant clue to understanding the impact of affective touch on body representation.

With a close look at the reviewed literature on RHI, body ownership and PpS share the great possibility to be measured through visuo-tactile paradigms. In particular, the spatiotemporal synchrony between visual and tactile stimuli is the essential prerequisite to capture body ownership by means of the RHI paradigm, as well as to measure PpS through VTI tasks. Given the current low replicability about the relation between body ownership and affective touch, as well as the lack of evidence on how PpS is shaped by affective touch, here we wanted to fill these gaps by carrying out two separate experiments. In the first one, the same pool of participants undertook a classical RHI paradigm in two different experimental sessions, where an affective (3 cm/s) or neutral (18 cm/s) touch was delivered during the entire task. To control for autonomic activity, we complemented the embodiment measures (i.e., proprioceptive drift and self-report scale) with electrodermal activity recording. In the second experiment, instead, a classical version of VTI task was administered twice to two different experimental groups, one receiving the affective touch (3 cm/s) and the other the neutral touch (18 cm/s) in the middle of the experimental session. Images of positive, negative, and neutral hands were used in order to investigate a possible role of the valence-connoted visual stimulus and its interaction with the valence-connoted touch. We expected to find stronger body ownership following affective rather than neutral touch, both at subjective (questionnaire) and objective (proprioceptive drift, SCL) level in and Experiment 1. In Experiment 2, we expected to replicate the robust facilitation effect for tactile stimuli when coupled with near visual ones, as well as to find a reduced spatial effect of visuo-tactile interaction after receiving affective compared to neutral touch because of the soothing effect of the former contact.

**Experiment 1. Affective Touch and Bodily Self.**

This experiment aimed to disentangle the ambiguous results already present in the existing literature on the influence of affective touch on body representation.

## 2. Materials and Methods

### 2.1. Participants

Fifty-four healthy subjects (48 females and 6 males, mean age 23.13 years, standard deviation 2.26, range: 20–30) participated in the experiment. All participants had a normal or corrected-to-normal vision. Except for three participants, all the others were right-handed. All students at the University of Milano-Bicocca received credits for their participation to the study and gave their written informed consent prior to performing the experiment. This research was performed in accordance with the Declaration of Helsinki and was approved by the local committee of the Department of Psychology, University of Milano-Bicocca (protocol number: RM-2017-112).

### 2.2. Procedure

Electrodermal Activity (EDA): Two Ag-AgCl electrodes with constant voltage (0.5 Volt) were attached to the participant’s distal phalanges of the right hand’s index and the middle finger. Electrodermal activity was recorded by means of a BIOPAC device (Biopac Systems Inc., Goleta, CA, USA) featuring a signal amplifier (MP150) and EDA data acquisition unit (GSR100C). Saline conductor gel was used to improve the signal-to-noise ratio. The gain parameter was set at 5 µS/Volt, with a sample rate of 100 Hz. The equipment was then calibrated according to BioPac system. For subsequent analysis, data were filtered offline using a high pass filter (0.05 Hz) to obtain phasic skin conductance responses (SCRs) related to the evoking stimuli [46]. A smoothing function with a sampling rate set at 50 KHz, equal to a half-second of recording, was administered to reduce the signal noise. Then, the numbers of phasic skin conductance responses (SCRs) that occurred during the stroking periods were identified as the of spontaneous fluctuations of the skin conductance beyond a set threshold of 0.02 μS [47,48]. In addition, the mean skin conductance level (SCL) during the two minutes was computed for each participant and stroking condition. Data were analyzed using the AcqKnowledge v4.2 software.

**Figure 1:**
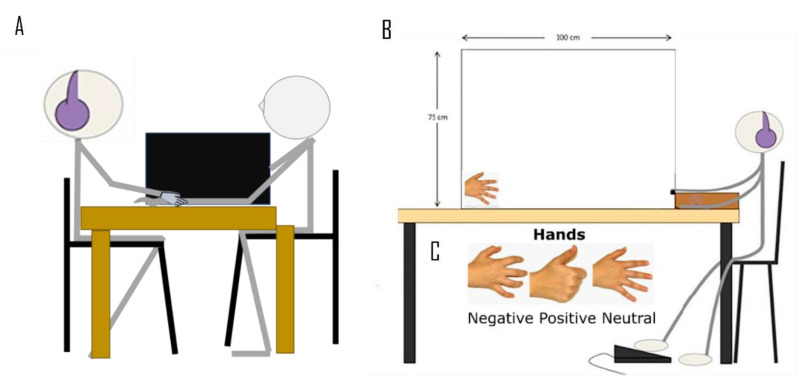
Sketch of the two experimental paradigms. (**A**) Rubber Hand Illusion paradigm administered in Experiment 1. The participant receives touches from the experimenter on both the rubber hand and the real hand with a slow (affective 3 cm/s) or fast (neutral 18 cm/s) stroking, either synchronous or asynchronous depending on the specific experimental condition. The participant is instructed to look at the rubber hand during the stimulation whose proximal edge was covered by a cloak; (**B**) Visuo-Tactile Interaction task administered in Experiment 2. The participant is seated in front of a table and looks at a looming visual stimulus while keeping attention on tactile stimulus that could be delivered when the visual one is located at 15, 30, 45, 60, 75, 90 cm from participant’s hand. The task is to respond as fast as possible to the tactile stimulus by pressing the foot pedal. The participant wears headphones, and a solenoid is attached to the right middle finger; (**C**) the three visual stimuli used in Experiment 2.

Rubber Hand Illusion Task. Participants were seated in front of a table with their left arm, palm down, placed in a black wooden box measuring 74 × 52 × 50, lacking in front and back sides, with the tip of their index finger resting on a fixed position marked on the table. The right hand was placed on the ipsilateral thigh so that it was not visible during the entire experiment and was kept still. First, participants were asked to estimate the location of the tip of their hidden index finger. The experimenter moved the cursor by sliding it on a ruler placed on the top side of the box, visible only to the experimenter, and asked participants to say “stop” at the point corresponding to the perceived location of their left index finger. The real location of participants’ left index finger was always matched to the 56° cm of the ruler, so that the difference between this actual position with the perceived one is an index of proprioceptive sensitivity, called proprioceptive drift [49]. This measurement was repeated four times by varying the starting position of the cursor in order to prevent from anchoring to the previous trial statement. Subsequently, participants were instructed to close their eyes so that the box could be removed, and the life-sized left rubber hand be placed on the table, following previously set coordinates, equal for all subjects (Figure 1—Panel A). 

A standing wooden partition board was also positioned beside the rubber hand, and a black cape was used to hide the proximal end of the rubber hand and participant’s left arm. The participant was instructed to gaze at the rubber hand while the experimenter strokes the rubber hand and the participant’s hidden left hand for 120 s concurrently with EDA recording. The duration of the induction phase was set at 120 s, according to the reported onset times for the illusion in the previous studies and the fact that all the participants were naive, experiencing the RHI for the first time [50,51]. The female experimenter used her index finger to stimulate an 18 cm extended skin portion of the dorsal part of the participants’ left forearm and the rubber hand. In order to limit participants’ CT fibers habituation, three different areas (i.e., lateral, radial, and medial) of the forearm were stroked. The stroking stimulation was applied to the forearm skin, where CT afferents are present and abundant [52,53]. The stimulation zone was delimited by an 18 × 1.5 cm paper plaster applied on the participants’ left forearm. Along this zone, three 3 cm-distant points were also marked to be used as references for providing the different velocity of tactile stimuli. Specifically, an affective touch (CT-optimal, 3 cm/s) or a neutral touch (CT-suboptimal, 18 cm/s) stimulation were administered to all participants. The experimenter wore earphones and was trained to use auditory signals from a metronome mobile application to control the rhythm of stroking velocity and to provide the stimulations at the correct rate. Moreover, throughout the duration of the experiment, the experimenter who delivered the stroking, took carefully under control sweating to avoid an increment of both the stroking/stroked hand temperature. Whenever noticing discomfort or fatigue sensations, experimenter was instructed to take a little break to restore the optimal testing conditions. For both neutral and affective touch, participants received synchronous and asynchronous stroking. To deliver synchronous stroking, tactile stimulation was simultaneously applied to the hidden participant’s real left hand and the rubber hand. Instead, asynchronous stroking entailed a temporal discrepancy between the tactile stimulation of the rubber hand and the participant’s left hand, so that the visible touch on the rubber hand did not spatially or temporally overlap with the perceived one on the left hand. After this stimulation period, participants were invited to close their eyes to allow experimenters changing the experimental setting and performing the proprioceptive drift measurement again. After that, a questionnaire of the embodiment experience (see below) was filled out. Participants underwent the four experimental conditions (two types of stroking velocity—Slow vs Fast-× 2 types of stroking mode—Synchronous vs. Asynchronous) in a randomized order. 

Embodiment Questionnaire. A 7-point Likert scale questionnaire (−3 strongly disagree; +3, strongly agree) was used to rate the subjective experience of Embodiment of the rubber hand, which was made up of three sub-components: Ownership—the feeling that the rubber hand is part of one’s body; Location—the feeling that the rubber hand and the real hand are in the same place; Agency—the feeling of being able to move the rubber hand. Other main components were also tested, such as Loss of own hand: the feeling of one’s hand disappearing and being out of one’s control and the feeling of being able to move one’s hand; Movement: related to the perceived motion of one’s hand and of the rubber hand; Affect: referring to the whole experience of the RHI being enjoyable [54]. Specifically, scores relative to one question of the Affect component evaluating the stroking pleasantness (22° item) were successively and independently used to differentiate the affective and neutral touch in terms of subjective pleasantness.

## 3. Results

**Figure 2:**
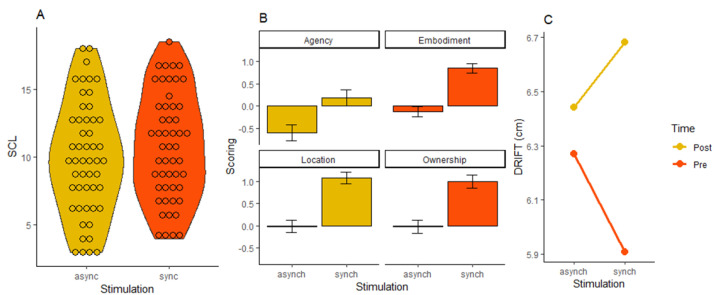
Results of Experiment 1. (**A**) Main effect of Stroking Mode for electrodermal activity. On the *x*-axis, the two levels of the variable Stroking Mode are represented while skin conductance level (SCL) is shown on the *y*-axis. Each point of the violin plot represents a subject; (**B**) Main effect of Stroking Mode for Embodiment Questionnaire. Participants showed an overall stronger body awareness while receiving the synchronous touch compared to the asynchronous one. On the *x*-axis, the two levels of the variable Stroking Mode are plotted while the scoring reported in the embodiment questionnaire is depicted on the *y*-axis. From the left top box to the right bottom one: Agency, Embodiment, Location, and Ownership. Error bars represent standard errors of the mean; (**C**) significant two-way interaction between Stroking Mode and Time for Proprioceptive Drift. The proprioceptive drift towards the rubber hand location was larger after the synchronous stroking than before it. On the *x*-axis, the two levels of the variable Stroking Mode are represented while proprioceptive drift (cm) is denoted on the *y*-axis.

EDA. A Repeated-Measures ANalysis Of VAriance (RM ANOVA) with Stroking Mode (synchronous vs. asynchronous) and Stroking Velocity (slow vs. fast) as within subject-factors related to SCL values was carried out to investigate the arousal response to the RHI experience. A significant main effect of Stroking Mode (F(1, 50) = 4.862; *p* = 0.032) was obtained. Bonferroni correction suggested that the synchronous stroking (M = 10.80, SD = 0.566) induced a higher number of phasic skin conductance responses (SCRs) than the asynchronous stroking (M = −0.601, SD = 1.310). No other significant effect was found (all *p* > 0.244) (Figure 2—Panel A).

Embodiment Questionnaire. Before submitting values to statistics, they were z-scores transformed. First, a paired sample *t*-test was conducted over the average stroking pleasantness ratings for Affective and Neutral Touch (22° item of the Affect component), but no significant result emerged (t(50) = 1.065; *p* = 0.292). To explore the likelihood of accepting the null hypothesis, we also performed a Bayesian paired sample *t*-test which consistently confirmed that affective and neutral touch were not differently perceived (BF01 = 3.846). RM ANOVAs were carried out with Stroking Mode (synchronous vs. asynchronous) and Stroking Velocity (slow vs. fast) as within subject-factors for all the (sub)components of the embodiment questionnaire. Results revealed a main effect of the factor Stroking Mode for the variable embodiment (F(1, 50) = 39.693; *p* < 0.001), as well as for its sub-components ownership (F(1, 50) = 29.687; *p* < 0.001), location (F(1, 50) = 35.084; *p* < 0.001), agency (F(1, 50) = 18.067; *p* < 0.001). Bonferroni correction indicated that, in all cases, the synchronous stroking (embodiment: M = 0.854, SD = 0.775; ownership: M = 0.996, SD = 1.043; location: M = 1.067, SD = 0.932; agency: M = 0.182, SD = 1.283) elicited a stronger feeling of body awareness than the asynchronous stroking (embodiment: M = −0.135, SD = 0.878; ownership: M = −0.016, SD = 1.019; location: M= −0.016, SD = 1.019; agency: M = −0.601, SD = 1.310). Stroking Mode turned out to be marginally significant also for the variable Loss of own hand (*p* = 0.052), but it did not reach significance for the variables Movement (all *p* > 0.285) and Affect (all *p* > 0.101). No other main effect or significant interaction was present (all *p* > 0.191) (Figure 2—Panel B).

Proprioceptive Drift. We calculated the final proprioceptive drift value for each participant by subtracting the difference between the actual and felt position of the left hand relative to the pre-session to that referring to the post-session. Then, a RM ANOVA with Stroking Mode (synchronous vs. asynchronous), Stroking Velocity (slow vs. fast) and Time (pre vs. post) as within subject-factors was carried out. The results revealed a main effect of Time (F(1, 50) = 7.136; *p* < 0.001) which was qualified by a significant two-way interaction between Time and Stroking Mode (F(1, 50) = 6.199; *p* = 0.016). Post-hoc test (Bonferroni) showed that, after receiving the synchronous touch, participants exhibited a stronger proprioceptive drift than before the stimulation (pre synchronous: M = 5.91, SD = 0.44; post synchronous: M = 6.68, SD = 0.46). This result means that the RHI worked since proprioceptive sense referred to the left hand was deviated towards the rubber hand location after the synchronous stimulation. No other effect reached significance (all *p* > 0.128) (Figure 2—Panel C).

## 4. Discussion

The present experiment aimed to clarify the effects of the gentle vs. neutral caress on body representation manipulated through the Rubber Hand Illusion. Along with replicating the strength of the illusion when tactile stimuli were delivered simultaneously to the fake and real hand, we found that the type of stroking is not a crucial factor in modulating our bodily self, both at behavioural and physiological level. 

Safe and pleasant touch is the basis of secure attachment, and it recalls earliest body memories. This is why, for instance, art therapies (e.g., the clay therapy) use haptic perception to cure traumas [55]. As stated in the introduction, more than one research work reported a link between pleasant touch and body representation, but a deeper look brings to light controversial findings. Indeed, an appellant problem of data replicability seems to emerge over these experiments as highlighted by the doubtful sensitivity of proprioceptive drift, embodiment questionnaire and electrodermal activity to the affective touch manipulation.

Although we confirmed the classical effect of the RHI on body representation through proprioceptive drift, embodiment questionnaire and SCR, all these measurements were not influenced by affective touch. Thus, our results enhance even further replicability problem on this topic since no effect of gentle touch on body representation has been detected. Importantly, we were also unable to find any significant difference in terms of self-reported touch pleasantness between the neutral and affective stroking as assessed by the specific question of the Affect component of the embodiment questionnaire. This result could be explained by the “skin to skin” contact between the experimenter and participant that we adopted in our study, which represents a novelty compared to the touch delivered by a soft brush implemented in previous studies on body representation [12,13,14,15,16,19,25,26,31]. Therefore, our data seems to suggest that the skin-to-skin inter-individual interaction overlapped to the soothing effect of the neutral touch, thus masking the typical effects of affective touch on body representation (see General Discussion for a more in-depth argumentation on this topic). Indeed, previous work has shown that active delivery of social touch to another human—without distinguishing between hairy and glabrous skin—enhances mu-opioid receptors’ availability in specific brain areas, a neurochemical mechanism reinforcing social bonds between individuals [56].

It is also true that inter-individual variability could have affected the null findings of our study. Indeed, it is well-known that inter-subject differences are certainly a crucial aspect when studying interoception, which is based on participant’s inner sense [57] and may become even more relevant in case of hedonic haptic contact [58,59]. Previous work has shown that other factors, such as autistic traits [18,60] (for a different perspective see [61,62]), motivational states [63], attachment style [64,65], sex [66], and behavioral inhibition system sensitivity [59] modulate affective touch. In support of this perspective, it is worth underlying that the general experience of touch—and not specifically the affective one—entails different brain responses among individuals even when tactile expectancy and attentional level are carefully taken under control [67]. Again, the rewarding system—which is intrinsically related to the hedonic sensation caused by affective touch—feels the effect of individual differences both at the neurobiological and genetic level [68,69]. In addition, the bodily illusion paradigm suffers from inter-individual differences too, such as sensory suggestibility [70], psychosis-proneness [71], empathy, and schizotypal traits [72].

Along with these critical points, the empirical evidence still lacks how the gentle caress could modulate the perception of the space intimately connected to our body, the peri-personal space, and we believe that new findings in this direction would help frame better the exact role of affective touch on bodily representation.

**Experiment 2. Affective Touch and Extra-Bodily Self.**

This experiment aimed to collect scientific evidence on the unexplored influence of affective touch on PpS representation.

## 5. Material and Methods

### 5.1. Participants

Forty-eight healthy subjects (15 males, mean age 23.73 years, standard deviation 2.69, range: 19–36, all right-handed) took part in the experiment. All participants had a normal or corrected-to-normal vision and intact sense of touch, as self-reported. They were all students at the University of Milano-Bicocca and gave their written informed consent prior to performing the experiment. They also received credits for their participation in the study. This research was performed in accordance with the Declaration of Helsinki and was approved by the local committee of the Department of Psychology, University of Milano-Bicocca (protocol number: RM-2017-112).

### 5.2. Procedure

Validation phase. In a pre-experimental validation session, a first group of participants (*n* = 40, 27 females, mean age ± SD = 23.5 ± 2.961 years, range = 19–33 years) was invited to assess 69 visual stimuli using a 9-point Likert scale. They had to rate two stimulus features: valence and arousal. After the stimulus presentation at the center of the screen, participants first evaluated the valence “Rate how negative/positive the picture just displayed is” and then the arousal “Rate how arousing the picture just displayed is” choosing among 9 alternatives (from “completely negative/relaxing” to “completely positive/exciting”, where the central point was the neutral point). The experimental visual stimuli were photos taken with a Canon Powershot SX40HS photo camera in a neutral setting. All pictures were taken from the same perspective and at the same distance of 27.5 cm. They included 25 negative, 30 positive, and 14 neutral images. This procedure allowed us to select the 3 images (positive hand, negative hand, neutral hand) used in the following experimental step (Figure 1—Panel C). Indeed, the three selected pictures characterized by the most relevant scores on each scale were put inside an RM ANOVA by separately analyzing valence and arousal scores. A significant main effect of Valence (F(2, 78) = 82.27; *p* < 0.001) was obtained. Post-hoc analysis (Tukey) revealed that positive hand had a significantly higher score than negative (*p* < 0.001) and neutral ones (*p* = 0.0028), as well as the negative hand was significantly lower rated than the neutral hand (*p* < 0.001) (positive hand: M ± SD = 5.950 ± 1.797; negative hand: M ± SD = 1.875 ± 1.223; neutral hand: M ± SD = 3.800 ± 1.843). The main effect of Arousal (F(2, 78) = 7.176; *p* < 0.001) was also recorded. Post-hoc analysis (Tukey) showed that the neutral stimulus was judged as less arousing than the negative (*p* < 0.001) and positive ones (*p* = 0.037), which in turn they did not differ between each other (*p* = 0.457) (positive hand: M ± SD = 4.900 ± 1.823; negative hand: M ± SD = 5.425 ± 2.049; neutral hand: M ± SD = 3.800 ± 1.843). This preliminary phase lasted about 10 min and was run by OpenSesame software v3.1 [73].

Visuo-Tactile Interaction Task. Participants sat in a barely illuminated room by positioning their righthand palm down on a table adjacent to the wall where visual stimuli were projected (Figure 1—Panel B). In agreement with the previous validation phase, approaching visual stimuli represent the three images of the positive, negative, and neutral hand (Figure 1—Panel C). Each trial reproduced a video where one out of three visual stimuli approach the participant’s hand, by travelling with a constant speed of 66 cm/s and covering an overall distance of 1 m. Along with the visual stimulus, in 85% of the trials, participants also received a tactile stimulation on the fingertip of their right middle finger. Their task was to press a foot pedal located under the table as soon as they perceived the tactile input. The remaining trials were catch trials (15%) in which no tactile stimulation was delivered, and any response was expected. Tactile stimuli were delivered at different fixed temporal delays from the visual stimulus onset corresponding to 6 different spatial positions (15, 30, 45, 60, 75 and 90 cm) of the approaching image for the participant’s hand. This experimental procedure was repeated twice, which is before and after the affective or neutral touch manipulation. The overall experiment consists of a random combination of 12 repetitions of each stimulus type for each spatial distance randomly intermingled with 36 catch trials. The inter trial interval was set at 500 ms. This led to a total of 252 experimental trials lasting 15 min, preceded by 1-min training where neutral stimuli (red balls) were presented as visual approaching stimuli, together with tactile stimuli, for 15 trials. Throughout the experiment, participants wore headphones delivering white noise to cover the light noise produced by tactile stimulators. Visual stimuli were included inside a 180 × 180 mm square and were projected in a 100 × 75 cm working area on the wall by means of a projector (Acer P7200i) connected to a computer (HP 6555b). All the animations were presented on a white background. The tactile pulse was a single pulse of 40 ms duration and consisted of a clearly perceivable ‘tap’. It was delivered through a little magnet within the solenoid (Heijo electronics, www.heijo.com (accessed on 7 January 2021)) that was attached to a participant’s fingertip and interfaced with the experimental computer by means of an ad-hoc built control relais-box (Tattile Box, s/n Touch15001, EMS, Bologna, Italy). The presentation of visual and tactile stimuli as well as the response collection were controlled by OpenSesame software v3.1 [73].

Touch manipulation. Immediately after completing the first VTI task, one group of participants performed the affective touch manipulation (*n* = 24) and the other one the neutral touch condition (*n* = 24). Participants were randomly assigned to one of the two groups. They were invited to put their right hand on the table in front of the experimenter, who began to stroke it with her right index finger (see pictures in Figure 2). The two groups, affective and neutral touch, differed for the velocity of stroking: the affective group received a slow touch (CT-optimal, 3 cm/s) and the neutral group a fast touch (CT-suboptimal, 18 cm/s). The stroke was delivered for 3 min in both conditions, and the most stimulated area of the participant’s right hand was the hairy skin between the thumb and the index finger. Experimenter, wearing headphones, used a metronome to keep stable the rhythm of stroking. Moreover, throughout the duration of the experiment, the experimenter carefully took under control sweating to avoid an increment of both the stroking/stroked hand temperature. Whenever the experimenters noticed discomfort or fatigue sensations, they were instructed to take a little break to restore the optimal testing conditions. Participants were instructed to look at the stimulated hand throughout the whole manipulation.

Questionnaire on touch pleasantness. Just after the affective or neutral touch manipulation, participants were requested to rate the pleasantness level of the received stimulation through a paper questionnaire made of the 9-point Likert Scale, which goes from “completely unpleasant” to “completely pleasant” where the central point indicates the neutral value. After filling out the questionnaire, participants were invited to repeat the VTI paradigm.

Questionnaire on hand touchability. At the end of the second session of the VTI task, participants were asked to indicate how they would like to get touched by the three hands observed during the VTI task. They had to respond by choosing one of the 9 Likert Scale points that go from “Not at all” to “Completely” in which the central point indicates the neutral value. The questionnaire and its response collection were controlled by OpenSesame software v3.1 [73].

## 6. Results

Touch Pleasantness. An independent samples *t*-test was carried out for Touch Pleasantness scores to verify whether the two groups (Affective Touch Groups vs. Neutral Touch Group) rated the received touch differently. Results indicated that there was no difference between the two groups (t(46) = −1.595; *p* = 0.118) (Affective Touch: M ± SD = 6.292 ± 1.628; Neutral Touch: M ± SD = 6.917 ± 1.018). Moreover, in line with Experiment 1, a Bayesian independent samples *t*-test on these values revealed that there is scarce evidence supporting the null hypothesis, which is the absence of a difference in terms of subjective pleasantness between the affective and neutral touch (BF01 = 1.248). 

Hand Touchability. Three participants were discarded from this analysis because of technical problems during data collection. The following analyses were thus conducted on 21 participants for the affective touch group and 24 participants for the neutral touch group. RM ANOVA on scores reported for Hand Touchability revealed a main effect of the within-subject factor Hand Valence (Positive, Negative, Neutral) (F(2, 88) = 42.349; *p* < 0.001). Bonferroni correction indicated that positive and neutral hands were judged as more touchable than the negative hand (both *p* < 0.001), while no difference was detected between positive and neutral hands (*p* = 0.999) (Positive Hand: M ± SD = 5.200 ± 1.517; Negative Hand: M ± SD = 2.889 ± 1.837; Neutral Hand: M ± SD = 5.489 ± 1.649).

Visuo-Tactile Interaction Task. For each participant, trials that exceed 3 SD from the mean within a specific experimental condition were discarded (4.68% trials were rejected overall). Both groups correctly performed the spatial task, as revealed by the presence vs. absence of responses to true and catch trials, respectively (Affective Pre: M ± SD = 93.6% ± 10.5%; Affective Post: M ± SD = 94.44% ± 6.9%; Neutral Pre: M ± SD = 90% ± 5.58%; Neutral Post: M ± SD = 94.56% ± 18.94%). Each participant responded with an accuracy > 75% in both sessions, therefore all subjects were included in subsequent analyses. The Shapiro–Wilk test for normal distribution revealed a significant difference (*p* < 0.05) for the great majority of variables, hence we decided to LOG transform our data before the analysis.

**Figure 3:**
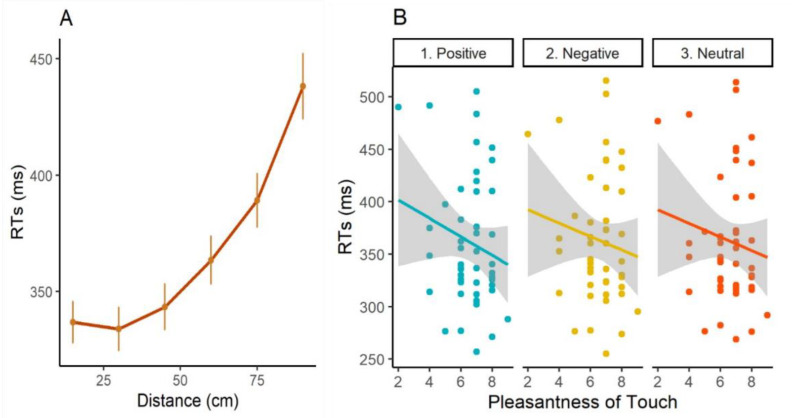
Results of Experiment 2. (**A**) main effect of distance. Participants were gradually faster to respond to the tactile stimulus as the visual one gets closer to the body. On the *x*-axis, the different distances in centimeters where the visual stimulus was located while tactile input was delivered on the right hand of participants. On the *y*-axis, RTs to the tactile stimulus in milliseconds. Error bars represent standard errors of the mean; (**B**) two-way interaction between Valence and Touch Pleasantness. Each point represents a participant. Positive valence is depicted in light blue, negative valence in yellow, and neutral valence in orange. As can be seen from the slope of the three trend lines, participants with lowest scores in Touch Pleasantness were slower to report the tactile stimulus (left-side) compared to participants with higher scores (right-side), and this difference seems to be particularly present when receiving visual stimuli connoted by positive valence. The different Likert Scale scores on Touch Pleasantness are reported on the *x*-axis while RTs in milliseconds are represented on the *y*-axis.

A first RM ANOVA was carried out with Distance (15, 30, 45, 60, 75, 90), Block (Pre, Post) and Valence (Positive, Negative, Neutral) as a within subject-factor, while the variable Group (Affective Touch, Neutral Touch) was used as between-subject factor. Results revealed a main effect of Distance (F(5, 230) = 162.582; *p* < 0.001). The post-hoc test (Bonferroni) showed that visual stimulus was not differently perceived when it was located at 15, 30, 45 cm from participant’s hand (all *p* < 0.255) while it was differently processed at 60, 75, 90 cm distances (all *p* < 0.001). In addition, all these three distances were significantly different among each other (all p < 0.001) (Figure 3—Panel A). There was also a main effect of Block (F(1, 46) = 12.080; *p* = 0.001), suggesting that, in the second session, participants were faster in responding to the tactile stimulus than in the first one. No other main effect or significant interaction has been reported (all *p* > 0.112). In order to understand whether the real pleasantness of touch could shape space perception, a second RM ANOVA was requested by adding in the first model the variable Touch Pleasantness as a covariate while the variable Group (Affective Touch, Neutral Touch) was removed as between-subject factor. Besides the main effect of Distance (F(5, 230) = 3.506; *p* = 0.004) that was not further analyzed, a main effect of Valence (F(2, 92) = 3.599; *p* = 0.031) followed by the two-way interaction between Valence and Touch Pleasantness were found (F(2, 92) = 4.760; *p* = 0.011). Participants with lower scores in touch pleasantness had slower RTs to tactile stimuli that were concurrently presented with positive visual stimulus, while the opposite was true for participants with higher scores in touch pleasantness (Figure 3—Panel B). No relevant difference seems to exist for negative and neutral valence. No other main effect or significant interaction was present (all *p* > 0.087).

Please note that, in both graphs, we used RTs’ raw values and not LOG-transformed ones as in the analysis.

## 7. Discussion

In the present experiment, we wanted to explore whether the space around our body is modulated by the gentle caress. Bearing in mind the soothing effect of the affective touch [14], a contraction of PpS would be expected following the soft stroking, while no changes with respect to the baseline should have emerged after the neutral touch. Contrary to our predictions, however, the different type of stroking—affective vs. neutral—did not have a different impact on the spatial multisensory integration. Overall, the response to tactile events was more efficient when they were coupled with the visual stimulus presented closer to the body rather than far away from it. Moreover, the degree of touch pleasantness—regardless of its affective or neutral valence—had an impact on the response to positive visual stimuli, given that individuals who rated the touch as more pleasant had faster reaction times to the tactile stimulus when it co-occurred with the positive hand compared to participants who experienced a less intense touch pleasantness. 

As mentioned in the introduction, PpS is well captured by the integration between visual and tactile stimuli. The way these multisensory interactions change in space following specific manipulations is commonly used as proof of a reduction/extension of PpS. Indeed, the more efficiently this integration is protracted in space, the farther the space would be classified as close [39,40]. Our results confirm this robust effect of spatial distance, which is reflected in faster reaction times to the tactile stimulus when the visual one is located closer rather than farther from the body. As suggested by [74], we agree that a sharp PpS boundary is unlikely, and embrace the idea of a gradual shift from peri- to extra-personal space. In line with previous findings, we found that significant RT changes typically occur in the interval between 45 and 60 cm [37,39,40].

Besides this effect, we also found a different role played by stimulus valence depending on the touch pleasantness experienced. In particular, the less pleasant the touch rating, the slower the reaction time to the positive image. In the same vein, the more pleasant the participants rated the haptic stimulation, the faster the response was to the positive hand in the visuo-tactile task. This could be compatible with the attenuation of negative affective responses by touch, as demonstrated by the reduced feeling of social exclusion [14] and the weaker negative facial emotions when experiencing the slow and pleasant rather than the fast touch [75]. In our study, such interaction did not emerge when considering the affective vs. neutral touch, but this is not surprising since the slow, gentle touch was not rated as significantly more pleasant than the fast one, as indicated by self-reporting measures. Even if this could be considered as a breaking point with respect to the previous literature [12,14,19,25,26,31], we replicated what we found in Experiment 1 where explicit and implicit measures were unable to qualify the slow touch as more pleasant than the neutral one. As suggested in paragraph 4, we believe that this missing difference could be explained by the “skin-to-skin” contact between the experimenter and participants embraced in this study, which strongly differs from the mediated touch typically delivered by a soft brush of previous studies (see General Discussion).

Recently, it was proposed that increased interoception is a likely mechanism driving changes in body schema, given that peripersonal stimuli are coded as extrapersonal stimuli after meditation [76]. This is entirely in line with another study, where PpS ‘boundary’ seems to be narrower for people with high rather than low interoceptive accuracy [77]. Our results extend these findings, by showing that a specific type of interoception, that is pleasantness deriving from social touch, is not capable of shaping PpS. Indeed, no difference in spatial perception emerged for individuals who rated pleasantness of the perceived touch as high or low. It is noteworthy that the absence of clues in the existing literature on how long the effect of affective touch lasts in time could be an explanation of the present null finding. Indeed, due to technical issues, a few minutes elapsed between the end of touch delivery and the beginning of the (second) spatial task. Moreover, we were also unable to find any effect of stimulus valence on PpS, contrary to our previous findings showing a stronger multisensory integration for valence-connoted stimuli than neutral ones [39]. However, it is worth underlying that, in this case, visual stimuli represent the images of real hands, and not inanimate objects as in [39]. Therefore, it is likely that, in the present experiment, interpersonal space—the space between individuals—rather than reaching or defensive spaces has been explored [78,79].

## 8. General Discussion

In the present study, we sought to find evidence on the role of affective touch inside and outside the bodily self. This aim arose from both the fragmented results present in the existing literature about the impact of a gentle caress on body representation [12,25,26,31], and the deficiency of empirical evidence supporting or not an influence of the soft touch on peripersonal space processing. By taking advantage of two well-established experimental tasks designed to tap into the bodily self (Rubber Hand Illusion Paradigm) and the extra-bodily self (Visuo-Tactile Interaction Task), we found that body ownership and peripersonal space representation are not at all affected by the interoceptive experience connected with the pleasant touch. Indeed, in Experiment 1, neither the classical RHI measurements—proprioceptive drift and embodiment questionnaire—nor the physiological one (SCR) show an alteration due to the different type of touch. Analogously, touch pleasantness is not able to diversely shape visuo-tactile interactions in space as reported in Experiment 2. Remarkably, any difference between the affective (slow) and neutral (fast) touch emerged in terms of touch pleasantness, as highlighted by physiological correlate (Experiment 1) and self-report measures (Experiments 1 and 2).

In particular, as described in the method session, we adopted a true “skin to skin” touch as opposed to the soft brush-mediated touch implemented by the great part of the existing literature investigating the relation between affective touch and body representation [12,13,14,15,16,19,25,26,31]. This means that, in our case, experimenter delivered touch to participants by directly stroking their fingers with her bare hand. In our opinion, this peculiarity of our experimental setting could be mainly responsible for the absence of a significant difference between the affective and neutral touch, for both SCR measures in Experiment 1 and explicit judgements of Experiment 2. Indeed, given the high efficacy of touch at therapeutic level [80] as well as the great emotion sensitivity linked to social touch [81], it is rational thinking that the real physical contact between experimenter and participant could have contributed to making the two touches very similar in terms of pleasantness. Importantly, Ref. [82] found that stroking others’ skin with the hand entails a greater activation in both primary and secondary somatosensory areas and posterior insula than stroking with a soft brush or any other material. They conclude that affective touch should be performed by avoiding inanimate objects’ mediation. The direct contact between experimenter and participants is thus the way to ensure ecological validity. Future research should promote the use of the real interpersonal contact when studying affective touch. The *social* affective touch cannot be replaced anymore in favour of an *asocial* (or partly social) affective touch. Indeed, as claimed in the introduction, the gentle caress has the final aim to promote interpersonal bonds with conspecifics [10] and not to feel pleasure for its own sake. Thus, the emotional connection with others established during the haptic contact needs to be appropriately considered when experimentally dealing with affective touch.

When developing our experimental paradigms, we did not believe that this approach could have represented such a confounding variable. Indeed, in its early days, the literature on affective touch was used to reproduce the gentle stroking through “skin-to-skin” contact [54,83,84,85]. However, in recent years, the scientific community has used a soft brush more to deliver affective touch without a strong reason for this choice. Indeed, neither force nor pressure of touch—which are two critical elements for activating CT-afferents [20,53]—can be finely controlled with a brush that is guided manually. Even if it is certainly true that a brush delivers a relatively constant force at a constant temperature, without needing a robot to apply the stroking, we believe that the very essence of gentle touch is sacrificed when stroking with a brush. In our everyday life, we often get in touch with other people by creating physical contact with them, and this relation is not mediated by any kind of tool. The appropriateness of the skin-to-skin contact seems to be also supported by [9] who demonstrated that human C-tactile afferents are tuned to the temperature of a skin-stroking caress. Authors showed that these fibers discharged preferentially to stimuli with a neutral (typical skin) temperature, rather than at the cooler or warmer stimulus temperatures. Thus, due to the neutral temperature inherent in the human skin, also for this reason, the skin-to-skin contact turns out to be the ideal approach to study the properties/effects of the pleasant touch.

To further entangle the experimental setting used for affective touch, it is worth underlying the absence of emotional connection between participants and experimenter. Indeed, there is not any prior knowledge in the touchant-touchè couple, and this feature could dampen the experimental outcome. In our case, for example, the lack of that surplus in participant’s pleasantness responses when receiving affective compared to neutral tactile stimulation could be partly attributed to the loss of that confidential relationship. Indeed, in everyday life, we enter into such physical contact with people that we adequately know, and this deep-rooted emotional connection may act as the nudge to trigger the most profound hedonic side of touch. Indeed, it has already been demonstrated that the type of relationship in the touching couple matters in both the topography and the effects related to affective touch [17,86]. Furthermore, a compromised efficacy of affective touch in people with pathological deficiencies in emotional interpersonal communication, like autistic individuals [7,61,87,88,89] (for a different perspective, see [61,62]) and anorexia nervosa patients [90], has been demonstrated.

### Limitations

Despite the new scientific evidence given by our study, it is not immune from some limitations. First of all, when selecting the neutral touch, we opted for a slower stroking (18 cm/s) (for a similar procedure, see: [14,17,23,25,26,90,91]) compared to that mostly used in the literature (30 cm/s) (for a similar procedure, see: [4,5,7,12,16,31,32,33,65] as the counterpart of the pleasant stimulation). The reduced experimental evidence about the haptic stimulation delivered at such velocity could have somehow impacted our data. It is also true that additional measures than the question we used for assessing the pleasantness of touch (e.g., Social Touch Questionnaire, Touch Experiences, and Attitudes Questionnaire) could have provided other precious information about the overall haptic experience in our participants. Moreover, the stimulation area and the timing of touch delivery did not overlap across the two tasks. Indeed, in Experiment 1, we focused on the forearm by stroking it for two minutes—in line with most studies dealing with RHI and affective touch [25,26]—while, in Experiment 2, we delivered a 3-min lasting touch on the right hand. This trick was used (*i*) to keep a constant spatial location of touch across the different experimental conditions and (*ii*) to extensively provide pleasant sensations that should have lasted throughout the duration of the visuo-tactile task. Also note that the stroking was delivered outside of the spatial task where the right hand was used as the reference for approaching visual stimuli as well as the target of the tactile input. Thus, we cannot completely exclude that the duration of stroking phase in Experiments 1 and 2 led to a possible slight increment of both the experimenter’s and participants’ hand temperature. Finally, our samples were not equally balanced by sex, and this could have impacted our results. Indeed, gender is a crucial factor in experiencing the pleasant sensations connected to the affective touch [66].

## 9. Conclusions

Beyond scientific production that seems to support the existence of a binding between body and pleasant touch [12,25,26,31], it comes as astoundingly natural to believe that pleasantness sensations could rearrange our corporeal and extra-corporeal space. Touching is such an intimate experience that requires an invasion of our private area [92]. However, in the present study, no difference in both the corporeal and extra-corporeal space depending on the diverse level of touch pleasantness has been found. For both RHI paradigm and the VTI task, we were unable to find any modulation of the pleasant touch on body representation and PpS perception. Notably, the ‘skin to skin’ contact occurred in our experimental setting, different from most previous studies and possibly causing the non-significant difference, both for explicit and implicit measurements, between affective and neutral touch conditions. Overall, our results suggest that, if differences among experimental conditions are more effectively induced through affective touch delivered by a paintbrush than a real human contact (that typically induces pleasant touch sensations in real social interactions), further research is needed to investigate the real nature of body representation and body/space interaction changes following experimental affective touch.

## Data Availability

Data are available on request.
